# Low Z‐4OHtam concentrations are associated with adverse clinical outcome among early stage premenopausal breast cancer patients treated with adjuvant tamoxifen

**DOI:** 10.1002/1878-0261.12865

**Published:** 2020-12-14

**Authors:** Thomas Helland, Bjørn Naume, Steinar Hustad, Ersilia Bifulco, Jan Terje Kvaløy, Anna Barbro Sætersdal, Marit Synnestvedt, Tone Hoel Lende, Bjørnar Gilje, Ingvil Mjaaland, Kjetil Weyde, Egil Støre Blix, Gro Wiedswang, Elin Borgen, Daniel Louis Hertz, Emiel Adrianus Maria Janssen, Gunnar Mellgren, Håvard Søiland

**Affiliations:** ^1^ Hormone Laboratory Department of Medical Biochemistry and Pharmacology Haukeland University Hospital Bergen Norway; ^2^ Department of Clinical Science University of Bergen Norway; ^3^ Department of Oncology Division of Cancer Medicine Oslo University Hospital Norway; ^4^ Institute of Clinical Medicine Faculty of Medicine University of Oslo Norway; ^5^ Core Facility for Metabolomics Department of Clinical Science University of Bergen Norway; ^6^ Department of Mathematics and Physics University of Stavanger Norway; ^7^ Department of Research Stavanger University Hospital Norway; ^8^ Department of Surgery Section for Breast and Endocrine Surgery Stavanger University Hospital Norway; ^9^ Department of Oncology and Radiotherapy Stavanger University Hospital Norway; ^10^ Department of Oncology Sykehuset Innlandet Gjøvik Norway; ^11^ Immunology Research Group Institute of Medical Biology University of Tromsø Norway; ^12^ Department of Oncology University Hospital of North Norway Tromsø Norway; ^13^ Department of GI‐Surgery Oslo University Hospital Norway; ^14^ Department of Pathology Oslo University Hospital Norway; ^15^ Department of Clinical Pharmacy University of Michigan College of Pharmacy Ann Arbor MI USA; ^16^ Department of Pathology Stavanger University Hospital Norway; ^17^ Department of Bioscience and Environmental Engineering University of Stavanger Norway

**Keywords:** 4OHtam, concentration threshold, endoxifen, outcomes, therapeutic drug monitoring

## Abstract

Low steady‐state levels of active tamoxifen metabolites have been associated with inferior treatment outcomes. In this retrospective analysis of 406 estrogen receptor‐positive breast cancer (BC) patients receiving adjuvant tamoxifen as initial treatment, we have associated our previously reported thresholds for the two active metabolites, Z‐endoxifen and Z‐4‐hydroxy‐tamoxifen (Z‐4OHtam), with treatment outcomes in an independent cohort of BC patients. Among all patients, metabolite levels did not affect survival. However, in the premenopausal subgroup receiving tamoxifen alone (*n* = 191) we confirmed an inferior BC ‐specific survival in patients with the previously described serum concentration threshold of Z‐4OHtam ≤ 3.26 nm (HR = 2.37, 95% CI = 1.02–5.48, *P* = 0.039). The ‘dose–response’ survival trend in patients categorized to ordinal concentration cut‐points of Z‐4OHtamoxifen (≤ 3.26, 3.27–8.13, > 8.13 nm) was also replicated (*P*‐trend log‐rank = 0.048). Z‐endoxifen was not associated with outcome. This is the first study to confirm the association between a published active tamoxifen metabolite threshold and BC outcome in an independent patient cohort. Premenopausal patients receiving 5‐year of tamoxifen alone may benefit from therapeutic drug monitoring to ensure tamoxifen effectiveness.

## Graphical abstract

Concentrations of active tamoxifen metabolites may be predictive of tamoxifen efficacy, and several therapeutic thresholds have been reported. This study of 406 ER+ breast cancer patients replicated an association between a previously reported active tamoxifen threshold (Z‐4OHtam ≤ 3.26 nm) and adverse BC outcome. The association was identified in early‐stage premenopausal patients using tamoxifen alone without sequential aromatase inhibitor use.

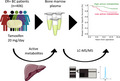

**Abbreviations**
BC: breast cancer
BCSS: breast cancer‐specific survival
95% CI: 95% confidence interval
ER: estrogen receptor
HER2: human epidermal growth factor receptor‐2
LC‐MS/MS: liquid chromatography‐mass spectrometry/ mass spectrometry
pN: pathological nodal status
pT: pathological tumor size
TDM: therapeutic drug monitoring
Z‐4OHtam: Z‐4‐hydroxy‐tamoxifen

## 1 Introduction

Endocrine therapy is indicated for estrogen receptor (ER)‐positive breast cancer (BC) patients to attenuate the cell supportive effect of estrogens and prevent growth of residual micro‐metastatic disease after surgery. This can be achieved by mitigated production of estrogens by aromatase inhibitors in postmenopausal women or by inhibiting ER transcriptional activity by the use of selective ER modulators like tamoxifen in premenopausal women. Tamoxifen may also be used in sequence with aromatase inhibitors for peri‐/postmenopausal women [1]. Adjuvant treatment with tamoxifen for 5 years significantly improves outcomes for women with ER‐positive BC [2], and extending the treatment time to 10 years adds additional outcome benefits [3].

Despite the improved outcomes, ~ 25% of patients using tamoxifen experience a relapse within 10 years [2]. Thus, micro‐metastases harbor endocrine refractory traits in these patients [4, 5], meaning that resistance to a particular antihormonal therapy has been developed. Several mechanisms of endocrine resistance have been identified including *ESR1* mutations and switch to ER‐independent estrogen‐induced signaling pathways [6, 7]. However, metabolic resistance may be an additional mechanism of resistance to tamoxifen.

Metabolic resistance to tamoxifen is the inability to form adequate active metabolite concentrations, thereby not achieving sufficient ER antagonism. Tamoxifen is regarded as a weak antiestrogen and is believed to be dependent on enzymatic biotransformation into the active metabolites 4‐hydroxy‐N‐desmethyl tamoxifen (Z‐endoxifen) and 4‐hydroxy‐tamoxifen (Z‐4OHtam) to reinforce its antiestrogenic effect [8, 9, 10]. These two metabolites have ~ 100× higher affinity to the ER compared with tamoxifen itself [10], and it is hypothesized that tamoxifen's therapeutic effect is dependent on adequate formation of these two metabolites. The hypothesis stems from a long line of evidence showing that activity impairing polymorphisms in the tamoxifen‐metabolizing enzyme CYP2D6 significantly reduce concentrations of endoxifen and 4OHtam in BC patients [11, 12, 13] and multiple studies have linked *CYP2D6* genetic variants with diminished metabolic activity to worse tamoxifen treatment outcome [14].

However, the validity of *CYP2D6* genotypes as a surrogate biomarker for systemic concentrations of active metabolites and thereby the clinical effect of tamoxifen remains unclear due to heterogeneous results [15]. As opposed to using a surrogate marker like *CYP2D6* that only explains portions of the variability in endoxifen concentrations, a more direct method for testing the hypothesis of metabolic resistance is to measure systemic levels of active metabolites and investigate their association with BC outcome. Three retrospective studies [12, 16, 17], including a recent publication by our group [17], have reported inferior BC outcomes in tamoxifen‐treated patients with low steady‐state levels of active tamoxifen metabolites. The opposite effect was reported in an explorative hypothesis‐generating study [18]. The association has not been validated prospectively [19, 20, 21] in various BC subgroups, and the clinical validity of the association still remains unclear [15, 22]. The inconsistent findings from many studies on the association of endoxifen levels or *CYP2D6* genotypes with tamoxifen treatment outcomes have resulted in conflicting clinical guidelines on whether to use any predictive biomarker to optimize tamoxifen treatment [23, 24, 25, 26]. There is therefore a need for evidence‐based guidelines regarding the possibility for therapeutic drug monitoring (TDM) of tamoxifen in BC patients. In this study, we aimed to provide further evidence on the association between impaired tamoxifen metabolism and BC outcome by confirming the predictive effect of our previously determined therapeutic concentration thresholds for Z‐4OHtam (3.26 nm) and Z‐endoxifen (9 nm) in an independent patient cohort.

## 2 Materials and methods

### 2.1 Patients

Patients included in this retrospective analysis were selected from subjects on a prospective trial investigating the value of Secondary Adjuvant (rescue) Treatment with docetaxel (Taxotere) [SATT (Secondary Adjuvant Treatment with Taxotere) study] for BC patients with disseminated tumor cells in their bone marrow (BM) [27]. The original study prospectively enrolled 1121 patients between October 2003 and May 2008 from seven Norwegian hospitals. Inclusion criteria were completion of primary surgery, completion of six cycles of anthracycline (AC)‐containing chemotherapy, and node‐positive or pT1c‐T4G2‐3 node‐negative BC with no earlier or other concomitant carcinoma. Endocrine treatment was given according to national guidelines at the time, which indicated 5 years of tamoxifen for ER+ patients included before 2005 regardless of menopausal status. From 2005, tamoxifen was given for 5 years to ER+ premenopausal patients and for 2–3 years followed by 3–2 years of an aromatase inhibitor for ER+ postmenopausal patients ≥ 55 years.

**Fig. 1:**
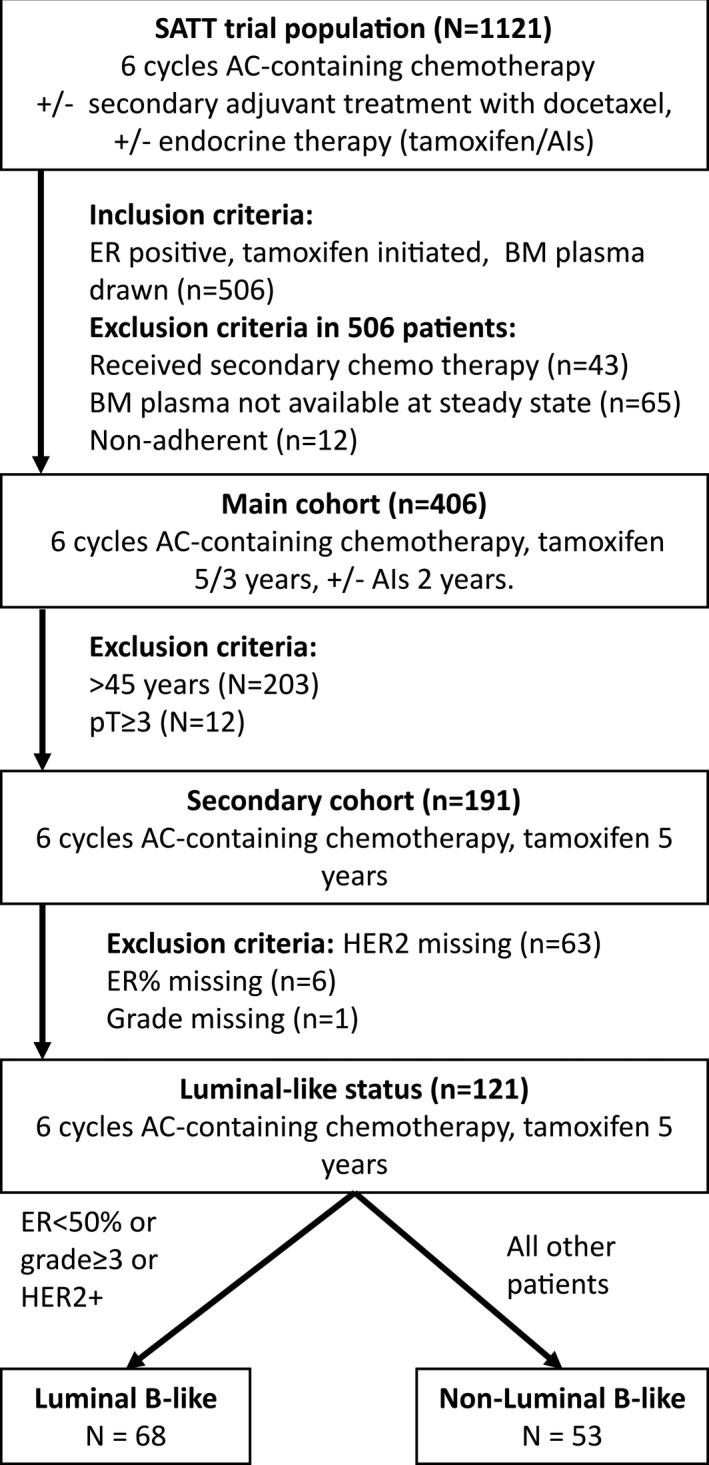
Patient flow from SATT trial into present analyses. Nonadherence represents patients with tamoxifen concentrations below 40 nm.

From this patient population, 406 ER+ patients who received tamoxifen, had BM plasma sample(s) available, and did not receive secondary adjuvant treatment with docetaxel (i.e., did not have detectable disseminated tumor cells in the BM 8–9 months after completing adjuvant chemotherapy) (Fig. 1) were included in this study. Thus, they reflect patients treated with endocrine treatment outside studies, according to national guidelines.

From the 406 patients, a ‘tamoxifen‐only’ subgroup including 191 operable patients < 45 years of age with a pathological tumor size (pT) ≤ 5 cm was selected. Selection of patients younger than 45 years ensured that the patients received tamoxifen as a monotherapy for 5 years according to guidelines at the time of inclusion [28]. The selection of pT1‐2 represents primary operable patients which ensured that neoadjuvant treatment had not been given, in line with the primary operable patient population from the learning set. Patients with plasma tamoxifen concentrations of < 40 nm were considered to be nonadherent to tamoxifen and excluded from both subgroups [12]. As molecular subtyping was not available, the relevance of luminal subgroup status on active metabolite thresholds was investigated by creation of a Luminal B‐like subgroup based on tumor pathological surrogate markers available. Information on Ki‐67 and PgR percentage was not available, and the following approximation was used to create the groups for this *post hoc* subgroup analysis; Luminal B‐like: ER level < 50% or grade ≥ 3 or human epidermal growth factor receptor‐2 (HER2) positive. All other patients were grouped as Luminal A [29, 30].

### 2.2 Extraction of bone marrow plasma

To investigate the prognostic value of disseminated tumor cells, 40 mL BM was aspirated from the posterior iliac crests bilaterally at two different time points after primary chemotherapy to isolate mononuclear cells. This procedure is described in detail elsewhere [31]. During the isolation of mononuclear cells, BM samples were diluted 1 : 2 in PBS and centrifuged 10 min at 1000 ***g***. After centrifugation, 3 × 1 mL samples of BM plasma was removed and stored at −40 °C and later transferred to −80 °C.

### 2.3 Quantification of tamoxifen metabolites in bone marrow plasma

Tamoxifen and seven metabolites (Table 2) were quantified in human BM plasma samples collected ~ 8 months from tamoxifen initiation using our previously described method for serum [17]. In brief, protein precipitation (20 μL) of BM plasma was performed in acetonitrile containing deuterated internal standards. The supernatant (80 μL) was evaporated to dryness using nitrogen and subsequently reconstituted in 500 μL water : methanol (20 : 80, v:v). Subsequently, the samples were chromatographically separated on a Waters Acquity UPLC system (Milford, MA, USA) using a Waters BEH Phenyl column (100 mm × 2.1 mm, 1.7 μm particle size). The column was developed by a gradient elution of 0.01% aqueous solution of formic acid and methanol as weak and strong mobile phases, respectively. The compounds were subjected to atmospheric pressure photoionization and detected in positive ion mode using a Xevo TQ‐S tandem mass spectrometer (Waters, Taunton, MA, USA). All measured concentrations were multiplied by 3 to account for the dilution factor caused by adding 2 volumes of PBS during the isolation of mononuclear cells.

### 2.4 Statistical approaches

The primary objective of this retrospective observational analysis of a prospective clinical trial was to confirm the predictive effect of our previously reported therapeutic thresholds for Z‐4OHtam (3.26 nm) and Z‐endoxifen (9.00 nm) on tamoxifen treatment outcomes [17]. The secondary objectives were to investigate the putative association of increasing survival trends or dose–response effects using our previously reported ordinal categories of low, moderate, and high concentrations of Z‐4OHtam (≤ 3.26, 3.27–8.13, > 8.13 nm) and Z‐endoxifen (≤ 9.00, 9.01–59.59, > 59.59 nm) [17].

Survival analyses included Kaplan–Meier and Cox regression analyses in which breast cancer‐specific survival (BCSS), defined as time from inclusion to death by BC, was used as the main outcome variable. The log‐rank test was applied to test for statistical difference in survival between groups. For Cox regression models, we report hazard rates (HR) with 95% CI and corresponding *P*‐values. First, we fitted univariable Cox models. Next, in multivariable Cox regression models we included both clinically relevant variables and variables with a *P*‐value ≤ 0.2 from the univariable analysis. Backward model selection and forward model selection were used to suggest the final multivariable model. Distribution of metabolite concentrations and clinical variables was calculated and presented as means, medians, and frequencies. Interpatient variability in tamoxifen concentrations was calculated as coefficients of variation. Ratios between Z‐endoxifen and Z‐4OHtam were calculated by dividing the two metabolite concentrations and reported as means with range. All statistical analyses were conducted using ibm ^®^ spss ^®^ Statistics version 25 (SPSS, Inc., Chicago, IL, USA). Two‐tailed *P*‐values < 0.05 were considered statistically significant.

## 3 Results

### 3.1 Patient characteristics

A total of 406 patients were eligible for this study. The clinical and demographic characteristics are presented in Table 1, and patient flow from the original trial population into secondary cohorts is presented in Fig. 1. All patients received adjuvant chemotherapy, and the majority (79%) of patients were premenopausal. The median follow‐up time for breast cancer‐specific death was 13.6 years. From this patient population, we selected the ‘tamoxifen‐only’ patient group (as described in Materials and methods) in order to study the tamoxifen metabolite effect on clinical outcome without potential influence from later change in endocrine treatment from tamoxifen to an aromatase inhibitor. This *a priori* determined subgroup had similar stage characteristics (pTpN) as the patients included in the learning set in which our thresholds were identified [17]. The median age of the ‘tamoxifen‐only’ group was 41 years, and the distribution of pT1 and pT2 tumors was close to 50/50 (Table 1).

**Table 1:** Patient characteristics. Study population, *N* = 406. ‘Tamoxifen‐only’ subgroup, *n* = 191. All patients received chemotherapy. Menopausal status was based on the information from case report forms within hospital records. PR, progesterone receptor; IDC, invasive ductal carcinoma; ILC, invasive lobular carcinoma.

Clinical/pathological features	Study population (*n* = 406)	‘Tamoxifen‐only’ subgroup (*n* = 191)
Follow‐up time BCSS, months, mean (median)	154.7 (163.2)	153.7 (163.3)
Age, years, mean (median)	43.7 (44.5)	40.1 (41.0)
< 45, *n* (%)	203 (50.0)	191 (100)
45–50, *n* (%)	202 (49.8)	–
50–55, *n* (%)	1 (0.2)	–
Menopausal status, *n* (%)		
Pre	322 (79.3)	166 (86.9)
Post	25 (6.2)	4 (2.1)
Missing	59 (14.5)	21 (11.0)
Surgery, *n* (%)		
Mastectomy	182 (44.8)	88 (46.1)
Lumpectomy	224 (55.2)	103 (53.9)
Tumor size (pT ), *n* (%)		
pT1	203 (50.0)	96 (50.3)
pT2	178 (43.8)	95 (49.7)
pT3	20 (4.9)	–
pT4	2 (0.5)	–
Missing	3 (0.7)	–
Grade, *n* (%)		
1	32 (7.9)	14 (7.3)
2	253 (62.3)	111 (58.1)
3	115 (28.3)	64 (33.5)
Missing	6 (1.5)	2 (1.0)
Histology, *n* (%)		
IDC	339 (83.5)	171 (89.5)
ILC	40 (9.9)	13 (6.8)
Missing	27 (6.7)	7 (3.7)
pN, *n* (%)		
Pos	249 (61.3)	115 (60.2)
Neg	157 (38.7)	76 (39.8)
HER2, *n* (%)		
Pos	33 (8.1)	15 (7.9)
Neg	234 (57.6)	113 (59.2)
Missing	139 (34.2)	63 (33.0)
ER, *n* (%)		
Pos	406 (100.0)	191 (100.0)
Neg	0 (0.0)	0 (0.0)
PR, *n* (%)		
Pos	350 (86.2)	165 (86.4)
Neg	50 (12.3)	24 (12.6)
Missing	6 (1.5)	2 (1.0)

### 3.2 Tamoxifen metabolite concentrations in bone marrow plasma

**Fig. 2:**
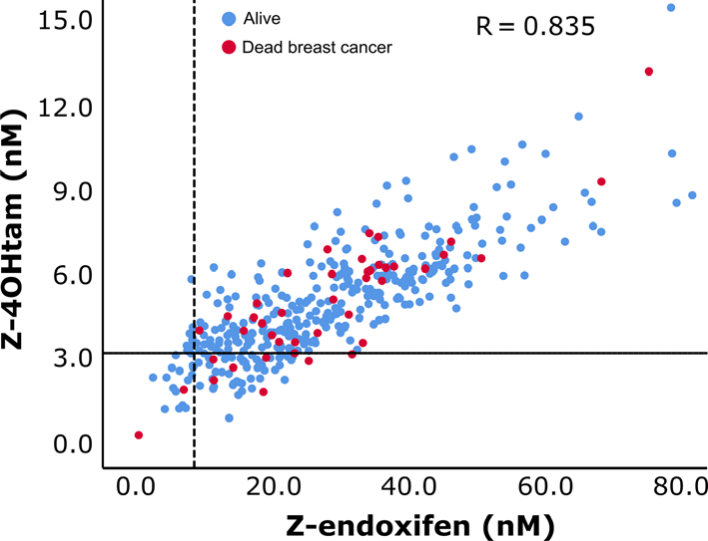
Distribution of Z‐endoxifen and Z‐4OHtam concentrations according to thresholds. Patients are from main cohort (*n* = 406). Reference lines on *y*‐axis and *x*‐axis represent 3.26 nm Z‐4OHtam (horizontal line) and 9.00 nm endoxifen thresholds (vertical line), respectively. Red circles represent patients dead of BC, and blue circles represent patients alive or dead of other causes.

Concentrations of tamoxifen and seven metabolites were measured in the BM plasma from 406 patients (Table 2). The same liquid chromatography‐mass spectrometry/ mass spectrometry (LC‐MS/MS) assay as for serum was applied with accuracy and precision well within the limits of requirements (Table S1). The mean and median concentrations found in BM were comparable to what has been measured in serum from other patient cohorts using the same LC‐MS/MS assay [17, 32]. This indicated that the concentrations measured in BM were representative of systemic tamoxifen metabolite concentrations. To investigate the systemic abundance differences (ratios) between Z‐endoxifen and Z‐4OHtam, the median ratio and range were calculated. For all analyzed patients (*n* = 406), the median was 5.7 (range 1.2–14.9) (Fig. S1). The ‘tamoxifen‐only’ subgroup had a median of 5.6 (range 1.9–14.9). In addition, a cross‐tabulation of the 191 patients grouped according to our previously reported cutoffs for Z‐4OHtam and Z‐endoxifen showed that 31% of the patients in the low Z‐4OHtam group belonged to the high Z‐endoxifen group (Fig. 2). Only 5.8% of the patients had combined low Z‐Endoxifen and low Z‐4OHtam.

**Table 2:** Tamoxifen metabolite concentrations. Concentrations are in nm. *N* = 406. CV, coefficient of variation (interpatient variability); Tam, tamoxifen; ND, n‐desmethyl; NNDD, N,N‐di‐desmethyl tamoxifen.

	Tam	NDtam	Z‐endoxifen	Z‐4'‐endoxifen	Z‐4OHtam	Z‐4'‐OHtam	TamNoX	NNDDtam
Mean	365.9	694.9	28.7	20.8	5.0	6.9	49.9	94.7
Median	341.2	655.8	26.6	19.6	4.8	6.6	43.1	89.7
CV	36.9	38.2	53.8	38.8	41.1	35.5	55.0	43.5

### 3.3 Tamoxifen metabolite concentrations in association with breast cancer outcome

**Fig. 3:**
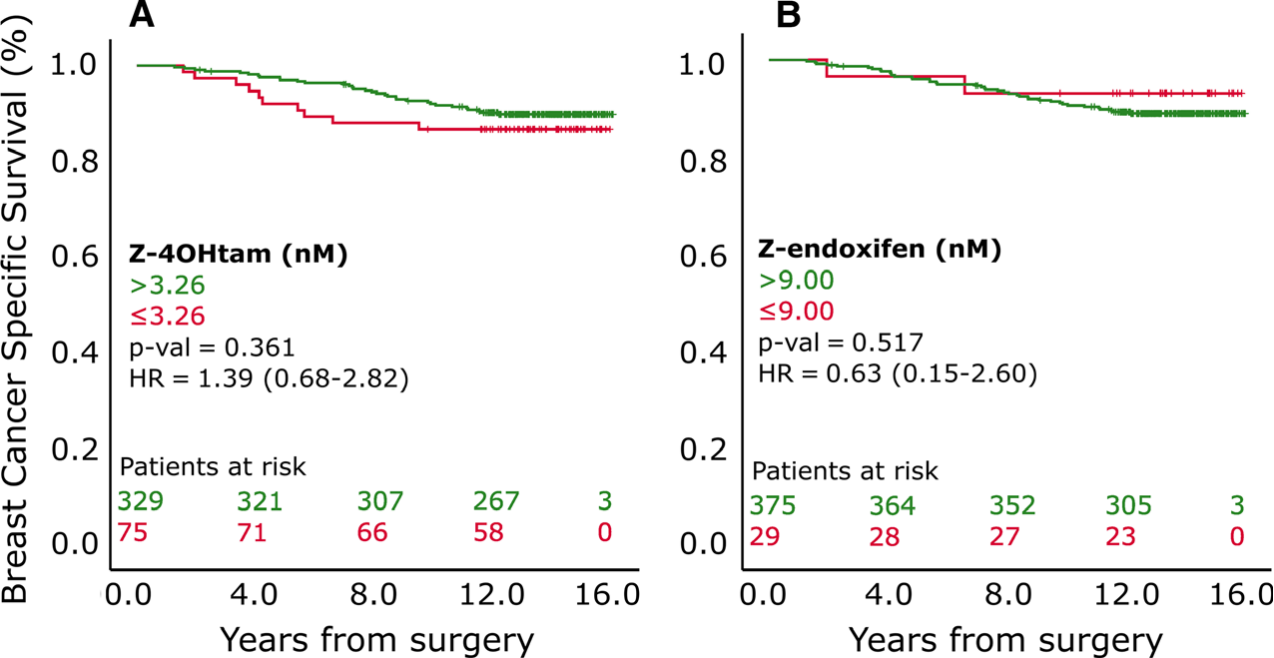
BCSS according to active metabolite thresholds in 406 patients. Panels A and B represent patients grouped according to Z‐4OHtam and Z‐endoxifen thresholds, respectively, in all patients included (*n* = 406). Log‐rank tests were used to determine differences in BCSS between groups. Patients at risk are shown according to color coding.

**Fig. 4:**
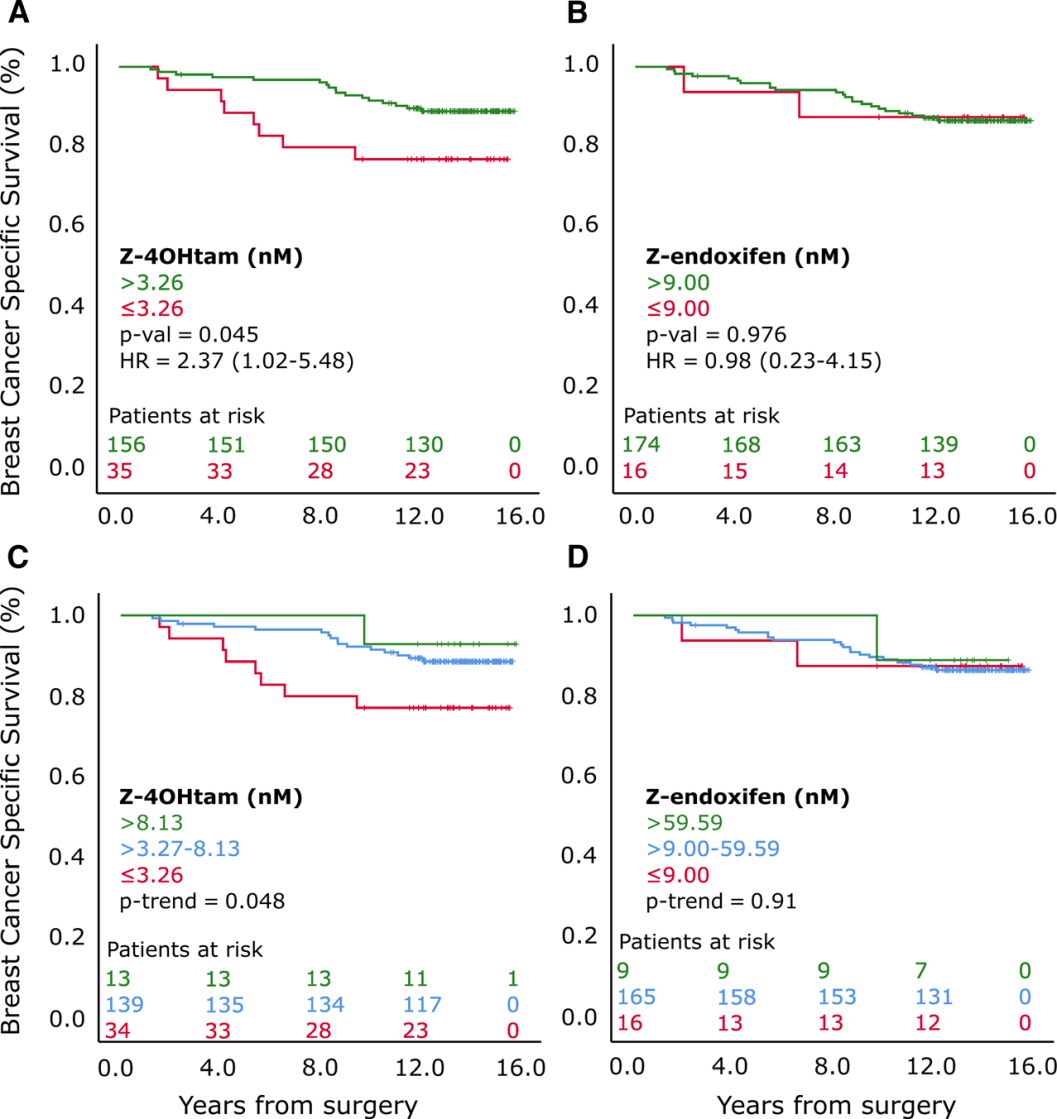
BCSS according to active metabolite thresholds in ‘tamoxifen‐only’ subgroup. Panels A and B represent patients grouped according to Z‐4OHtam and Z‐endoxifen thresholds, respectively, in ‘tamoxifen‐only’ subgroup (*n* = 191). C and D are patients grouped according to ordinal Z‐4OHtam and Z‐endoxifen levels, respectively, in ‘tamoxifen‐only’ subgroup. Log‐rank tests were used to determine differences in BCSS between groups. Patients at risk are shown according to color coding.

To investigate the predictive value of Z‐endoxifen and Z‐4OHtam thresholds of 9 and 3.26 nm, respectively [17], the concentrations were split above and below these thresholds and uni‐ and multivariable survival analyses were performed. Z‐endoxifen and Z‐4OHtam concentrations below the thresholds were not associated with BCSS in all 406 patients (Fig. 3A,B). The thresholds were next tested among the ‘tamoxifen‐only’ patients. In this subgroup, patients with Z‐4OHtam > 3.26 nm had improved survival compared with the ≤ 3.26 nm group (HR = 2.37, 95% CI = 1.02–5.48, *P* = 0.039; Fig. 4A). After adjusting for clinically relevant variables (Table 3), only Z‐4OHtam and pathological nodal status (pN) status were associated with BCSS in the final model with a HR of 2.69 (95% CI = 1.15–6.25) and 3.06 (95% CI = 1.14−8.19), respectively. No uni‐/multivariable associations were identified for the 9 nm Z‐endoxifen cutoff in this subgroup (Fig. 4B, Table 3).

**Table 3:** Univariable and multivariable analyses in secondary cohort (*n* = 191). PR, progesterone receptor; Z‐4OHtam, Z‐4‐hydroxy‐tamoxifen.

Factor	Event/patients	Survival %	HR (95% CI)	*P* (Cox)
Age (linear)	–		0.95 (0.87–1.03)	0.198
pT				
pT1	9/96	90.6%	1	
pT2	16/95	83.2%	1.89 (0.84–4.28)	0.127
pN				
Neg	5/76	93.4%	1	
Pos	20/115	82.6%	2.77 (1.04–7.38)	0.042
Pos^a^			3.06 (1.14–8.19)	0.026^a^
Grade				
Grades 1&2	15/125	88.0%	1	
Grade 3	10/64	84.4%	1.33 (0.60–2.97)	0.480
HER2				
Neg	13/113	88.5%	1	
Pos	2/15	86.7%	1.20 (0.27–5.31)	0.813
PR				
Pos	19/165	88.5%	1	
Neg	6/24	75.0%	2.69 (1.07–6.73)	0.035
Z–Endoxifen				
> 9 nm	23/174	87.5%	1	
≤ 9 nm	2/16	86.8%	0.98 (0.23–4.15)	0.976
Z4OHtam				
> 3.26 nm	17/156	89.1%	1	
≤ 3.26 nm	8/35	77.1%	2.37 (1.02–5.48)	0.045
≤ 3.26 nm ^a^			2.71 (1.15–6.25)	0.022^a^

^a^ Multivariable analyses with clinically relevant variables or variables with *P*‐value < 0.2 from the univariate analysis, that is, Z‐4OHtam, PR, age, pT, and pN in the model).

As part of the secondary objective, the previously reported ordinal categories of low, moderate, and high concentrations of Z‐endoxifen and Z‐4OHtam [17] were also compared with clinical outcome. A significant trend for increasing Z‐4OHtam concentrations and improved BCSS was identified for the ‘tamoxifen‐only’ subgroup (*P*‐trend = 0.048; Fig. 4C). No significant associations between ordinal categories of Z‐endoxifen and BCSS were identified (Fig. 4D).

As ER+ patients can be subgrouped into Luminal A and Luminal B subtypes with distinct biologically characteristics, the clinical relevance of low active metabolite concentrations in luminal surrogate subgroups of BC was explored in a *post hoc* analysis. Patients with and without Luminal B‐like features were created using surrogate markers as described in the Methods section. The 3.26 and 9 nm thresholds for Z‐4OHtam and Z‐endoxifen, respectively, were not predictive of survival in either subgroup (Fig. S2).

## 4 Discussion

In the present study, premenopausal patients with operable BC receiving 5 years of adjuvant tamoxifen without subsequent use of aromatase inhibitor had an inferior BCSS if serum concentrations of Z‐4OHtam were ≤ 3.26 nm. This is consistent with our findings from an independent patient cohort [17]. Moreover, we replicated our previously reported dose–response trend for enhanced BCSS using three ordinal groupings of Z‐4OHtam (≤ 3.26 nm, 3.27–8.13 nm, and > 8.13 nm) [17]. However, we were not able to identify an association with BCSS for our previously reported threshold Z‐endoxifen ≤ 9 nm.

When applying the thresholds to all 406 patients, we were not able to identify a predictive effect of the two active metabolites (Fig. 1A,B). Importantly, the tamoxifen treatment duration and potential inclusion of an aromatase inhibitor during the adjuvant endocrine treatment course differed between the entire study population in the present study and the patients included in the learning set [17]. In the learning set, all patients were recommended 5 years of tamoxifen, while in the current study 53% of the 406 patients may have switched to an aromatase inhibitor. Two separate retrospective studies have reported positive associations between active metabolites and outcome. These studies exclusively included patients receiving tamoxifen monotherapy [12, 16].

Further, the learning set consisted of patients with operable BC (pT1‐2), while the present main cohort (*n* = 406) included patients both with higher stage disease and endocrine treatment biases. Because of these differences, the patient selection for the present ‘tamoxifen‐only’ subgroup (*n* = 191) was made to align with the stage characteristics of the learning set, consequently reducing the heterogeneity within the patient population. The International Tamoxifen Pharmacogenomics Consortium performed a meta‐analysis in 4973 patients from 12 globally distributed study sites [14], and a considerable heterogeneity among patients from the various study sites was reported. When strict eligibility requirements were applied, poor metabolizer status on *CYP2D6*‐level was significantly associated with poorer survival (HR = 1.25; 95% CI = 1.06–1.47). However, this pattern was blurred when the inclusion criteria were not specified. Thus, studies allowing for robust and strict inclusion criteria were advocated. This is in line with our results, showing the importance of selecting homogenous groups when investigating the association between tamoxifen metabolism and outcome. However, the meta‐analysis was criticized for testing many potential subgroups and the important point of whether the subgroups were determined *a priori* was raised [33]. In the present study, the ‘tamoxifen‐only’ subgroup analysis was determined *a priori*.

No association was observed between level of active metabolites and luminal‐like subtypes, but the results of this *post hoc* analysis should be interpreted with caution both because of the low number of patients and the proxy‐determination of subtype that differ from the PAM‐50 luminal subtypes. Previous research indicates that patients with PAM‐50 determined luminal B status may benefit more by achieving threshold active metabolite concentrations [34] compared with Luminal A.

The lack of predictive effect of Z‐endoxifen is in contrast to other retrospective studies investigating tamoxifen metabolites in association with outcome [12, 16]. These thresholds were not associated with BCSS in our dataset. Kaplan–Meier analyses using the threshold from Madlensky (16 nm) and Saladores (14.15 nm) resulted in log‐rank *P*‐values of 0.349 and 0.523 for the main cohort and 0.710 and 0.754 for the ‘tamoxifen‐only’ cohort. The significance of Z‐4OHtam over Z‐endoxifen in this dataset may be attributed to the number of unique patients captured by the thresholds of Z‐4OHtam and Z‐endoxifen. A cross‐tabulation between the Z‐4OHtam and Z‐Endoxifen thresholds demonstrated that only 5.8% of the patients had combined low Z‐endoxifen and Z‐4OHtam concentrations. Further, 68% of the patients within the low Z‐4OHtam threshold were above the Z‐endoxifen threshold. In addition, in this dataset the Z‐4OHtam threshold captured twice as many patients below the threshold (*n* = 34) compared with patients found below the Z‐endoxifen threshold (*n* = 16) (Fig. 2). Our prior report on these thresholds yielded hazard ratios (HR) of 3.56 and 3.73 for Z‐4OHtam and Z‐endoxifen, respectively [17]. *Post hoc* power calculations using these HRs show that there was 89% and 61% chance of detecting a significant difference between the Z‐4OHtam and Z‐endoxifen groups, respectively, with the sample sizes we have in the current study. Using a more realistic HR of 2.5, the power calculations resulted in 65% and 34% power to detect differences between the groups. Thus, in regard to the Z‐endoxifen threshold this study was underpowered to detect a meaningful difference in survival and studies including more patients should be conducted to further elucidate the potential effect of the Z‐endoxifen threshold. Z‐4OHtam and Z‐endoxifen have the same affinity to the ER (100× compared to tamoxifen); however, Z‐endoxifen has been reported to be up to 10 times more abundant in serum compared with Z‐4OHtam and has therefore been considered the main effector in terms of ER blocking. As a measure of abundance, we report that the median ratio between the two active metabolites is 5.2 and ratios as low as 1 : 2 were identified. The ratio was not normal distributed, but rather skewed toward the lower values (Fig. S1), indicating that Z‐4OHtam may be more abundant and of higher clinical relevance than previously noted.

There are limitations to this study. First, our study used BM plasma to measure systemic tamoxifen metabolite concentrations. Although the concentrations we measured were highly comparable to previous LC‐MS/MS tamoxifen metabolite concentrations [17, 32], and research on drug concentration ratios between systemic serous fluids and BM plasma [35] indicates highly similar concentrations as in serum, the optimal approach would have been to compare parallel serum and BM plasma samples from the same patients. However, serum samples were not available.

A second weakness was the relatively low number of patients (191 out of 406) that were part of the ‘tamoxifen‐only’ subgroup in which the predictive effect of Z‐4OHtam was replicated. However, the median follow‐up time was 15.5 years, which ensured enough events for survival analysis.

An additional weakness includes not having data on adherence or concomitant drugs that may act as inhibitors or inducers of different tamoxifen‐metabolizing enzymes. Patients may have become nonadherent or initiated concomitant drugs that interact with tamoxifen after the timepoint at which the metabolites were measured.

The strengths of this study include a highly sensitive, selective, and reproducible detection method for active tamoxifen metabolites, a homogenous patient cohort treated with adjuvant tamoxifen alone and a long follow‐up time allowing for use of BC ‐specific survival as endpoint.

## 5 Conclusions

In conclusion, this study confirmed the association of our previously determined therapeutic Z‐4OHtam threshold (≤ 3.26 nm) with inferior tamoxifen treatment outcomes in early‐stage BC patients receiving tamoxifen monotherapy for 5 years. This gives hope for TDM as a principle to personalize and optimize treatment. However, additional validation and testing of clinical utility is needed in light of recent conflicting results on the association between tamoxifen metabolism and BC outcome [22]. If supported by future studies, this could give the clinicians the opportunity to adjust the patient's dosing regimen based on drug concentration measurements, avoiding undertreatment, and inferior clinical outcome. For patients who do not reach the threshold after dose escalation, a switch to an alternative endocrine treatment could be advised. Our results suggest that Z‐4OHtam is a metabolite candidate for such use and could be helpful for further improvement in long‐term survival of ER+ BC patients.

## Conflict of interest

The authors declare no conflict of interest.

## Author contributions

TH participated in planning, obtaining funding, analyzing data, and drafted the manuscript. BN was the PI of the original trial and ensured access to clinical samples and data. BN also participated in analysis of data and writing of the manuscript. SH and EB performed all work related to the LC‐MS/MS measurements. JTK performed the statistical analyses. ABS, MS THL, BG, IM, KW, ESB, and GW were responsible patient enrollment, collection, and assembly of data in the original trial. EB performed the pathological analyses. DLH and EAMJ contributed in interpretation of data and critical reviewing of the manuscript. GM and HS participated in planning, obtaining funding, analyzing data, and drafting of the manuscript. All authors approved the final version of the manuscript.

## Ethics approval and consent to participate

The SATT study was approved by the Regional Ethical Committee (reference S‐03032). The current substudy was described in Amendment 5 of the protocol and approved by the Regional Ethical Committee in 2017 (reference 2011/396).

### Peer Review

The peer review history for this article is available at https://publons.com/publon/10.1002/1878‐0261.12865.

## Supporting information


**Table S1.** Accuracy and precision for tamoxifen metabolites measured by LC‐MS/MS.

**Fig. S1.** Distribution of mean Z‐endoxifen and Z‐4OHtam ratios.

**Fig. S2.** Breast cancer specific survival according to active metabolite thresholds stratified by luminal‐like status.

## Data Availability

The datasets generated and/or analyzed during the current study are not publicly available due regulation regarding individual privacy, but are available from the corresponding author on reasonable request.
