# Supplementary material for: Low Z‐4OHtam concentrations are associated with adverse clinical outcome among early stage premenopausal breast cancer patients treated with adjuvant tamoxifen
Source: Mol Oncol. 2020 Dec 14;15(4):957–67. doi: 10.1002/1878-0261.12865 (PMC8024735; doi:10.1002/1878-0261.12865)
Supplement: Supplementary file 1 — Table S1. Accuracy and precision for tamoxifen metabolites measured by LC‐MS/MS. [file MOL2-15-957-s003.docx]

| **Analyte** | **Calibrator** | **Concentration** | **Total CV, %** | **Accuracy, %** |
| --- | --- | --- | --- | --- |
| Tamoxifen | Low | 29.6 | 4.4 | 98.6 |
|  | Medium | 88.7 | 3.1 | 100.4 |
|  | High | 266.2 | 2.9 | 102.3 |
| Tam-N-ox | Low | 7.3 | 8.7 | 96.1 |
|  | Medium | 21.9 | 5.1 | 98.5 |
|  | High | 65.8 | 6.2 | 101.8 |
| Z-4’Endoxifen | Low | 9.7 | 8.1 | 96.9 |
|  | Medium | 29.2 | 4.3 | 97.9 |
|  | High | 87.5 | 5.2 | 101.6 |
| Z-Endoxifen | Low | 4.2 | 13.2 | 93.2 |
|  | Medium | 12.5 | 6.7 | 96.4 |
|  | High | 37.4 | 5.8 | 103.2 |
| NDtam | Low | 59.3 | 8.1 | 97.5 |
|  | Medium | 177.9 | 4.2 | 100.4 |
|  | High | 533.6 | 2.9 | 102.8 |
| NNDDtam | Low | 14.7 | 13.3 | 93.2 |
|  | Medium | 44.1 | 6.0 | 99.0 |
|  | High | 132.4 | 5.9 | 101.9 |
| 4'OHtam | Low | 0.6 | 15.4 | 95.3 |
|  | Medium | 1.9 | 9.9 | 96.4 |
|  | High | 5.8 | 4.6 | 100.1 |
| Z-4OHtam | Low | 0.7 | 6.5 | 97.9 |
|  | Medium | 2.1 | 5.1 | 96.7 |
|  | High | 6.4 | 4.3 | 103.7 |

**Supplemental table 1. Accuracy and precision for tamoxifen metabolites measured by LC-MS/MS.** Concentrations are in nM. CV and accuracy based on inter-day variations between calibrators (QCs).
