# Supplementary figures and images for: Low Z‐4OHtam concentrations are associated with adverse clinical outcome among early stage premenopausal breast cancer patients treated with adjuvant tamoxifen
Source: Mol Oncol. 2020 Dec 14;15(4):957–67. doi: 10.1002/1878-0261.12865 (PMC8024735; doi:10.1002/1878-0261.12865)

**
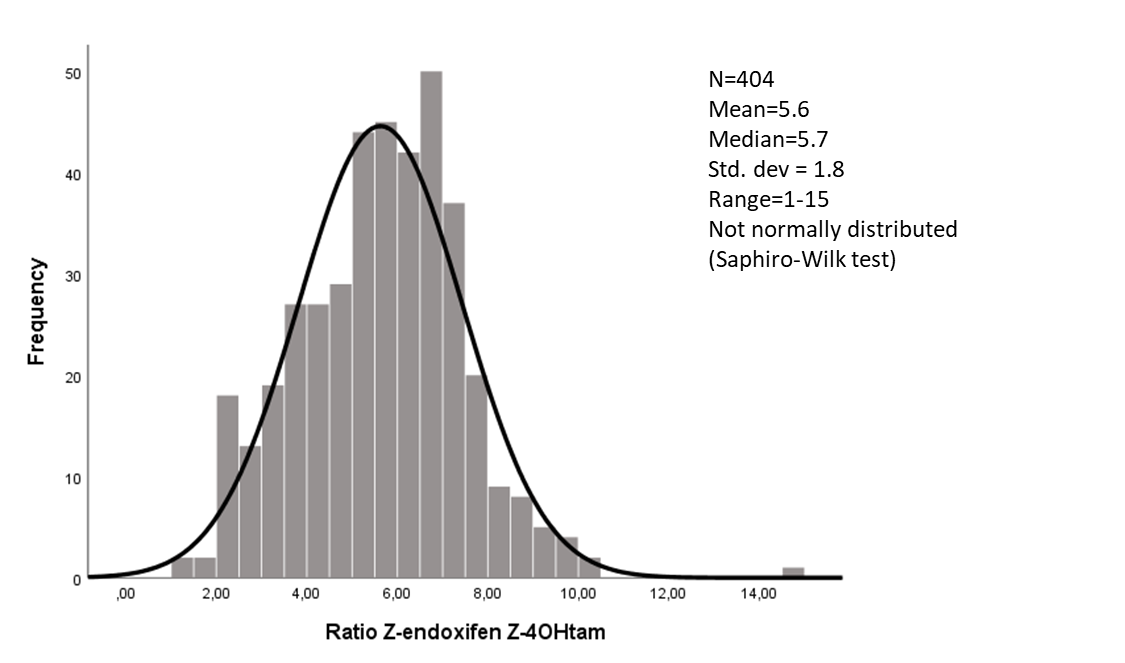
**

**Figure S1. Distribution of mean Z-endoxifen-Z-4OHtam ratio.**

Supplement: Supplementary file 2 — Fig. S1. Distribution of mean Z‐endoxifen and Z‐4OHtam ratios. [file MOL2-15-957-s001.docx]
