# Supplementary material for: Low Z‐4OHtam concentrations are associated with adverse clinical outcome among early stage premenopausal breast cancer patients treated with adjuvant tamoxifen
Source: Mol Oncol. 2020 Dec 14;15(4):957–67. doi: 10.1002/1878-0261.12865 (PMC8024735; doi:10.1002/1878-0261.12865)
Supplement: Supplementary file 3 — Fig. S2. Breast cancer specific survival according to active metabolite thresholds stratified by luminal‐like status. [file MOL2-15-957-s002.docx]

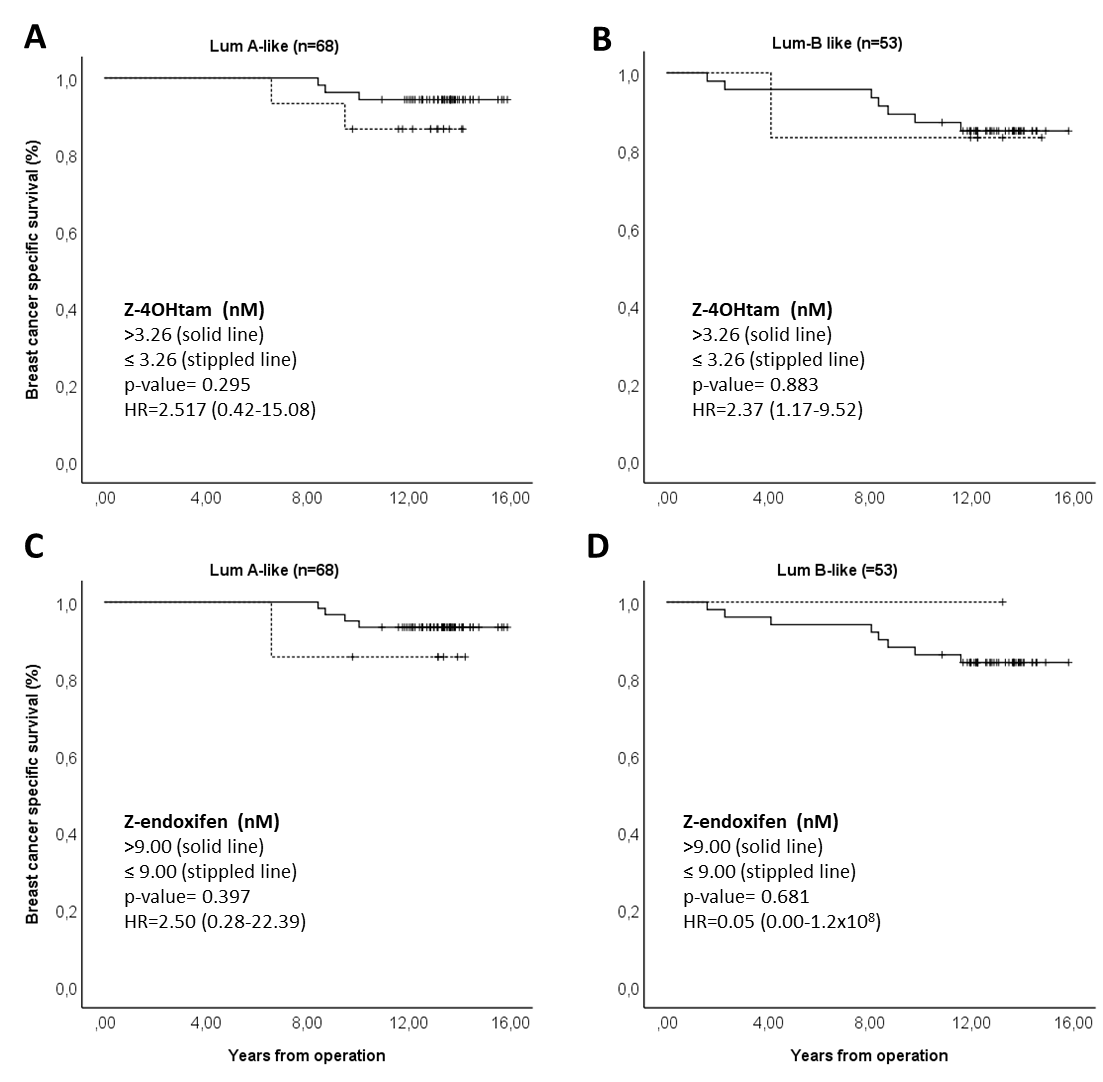


**Figure S2. Breast cancer specific survival according to active metabolite thresholds stratified by luminal-like status.** Luminal-like status based on ER level, HER2 and grade. Z-4OHtam ≤/> 3.26 nM threshold stratified by A) lum A-like and B) Lum B-like. Z-endoxifen ≤/> 9 nM threshold stratified by C) lum A-like and D) Lum B-like. Log-Rank tests were used to determine differences in breast cancer specific survival between groups. nM=nano molar, HR=hazard ratio, CI95=95% confidence interval.
